# Detection and classification of the integrative conjugative elements of *Lactococcus lactis*

**DOI:** 10.1186/s12864-024-10255-9

**Published:** 2024-04-01

**Authors:** Simon van der Els, Reshtrie Sheombarsing, Thijn van Kempen, Michiel Wels, Jos Boekhorst, Peter A. Bron, Michiel Kleerebezem

**Affiliations:** 1 Host-Microbe Interactomics Group, Department of Animal Sciences, Wageningen University & Research, De Elst 1, 6708 WD, Wageningen, The Netherlands; 2 NIZO B.V, Kernhemseweg 2, 6718 ZB Ede, The Netherlands; 3 BE-Basic Foundation, Mijnbouwstraat 120, 2628 RX, Delft, The Netherlands

**Keywords:** *Lactococcus lactis*, Mobile genetic elements, Integrative conjugative element (ICE), Comparative genomics

## Abstract

*Lactococcus lactis* is widely applied by the dairy industry for the fermentation of milk into products such as cheese. Adaptation of *L. lactis* to the dairy environment often depends on functions encoded by mobile genetic elements (MGEs) such as plasmids. Other *L. lactis* MGEs that contribute to industrially relevant traits like antimicrobial production and carbohydrate utilization capacities belong to the integrative conjugative elements (ICE). Here we investigate the prevalence of ICEs in *L. lactis* using an automated search engine that detects colocalized, ICE-associated core-functions (involved in conjugation or mobilization) in lactococcal genomes. This approach enabled the detection of 36 candidate-ICEs in 69 *L. lactis* genomes. By phylogenetic analysis of conserved protein functions encoded in all lactococcal ICEs, these 36 ICEs could be classified in three main ICE-families that encompass 7 distinguishable ICE-integrases and are characterized by apparent modular-exchangeability and plasticity. Finally, we demonstrate that phylogenetic analysis of the conjugation-associated VirB4 ATPase function differentiates ICE- and plasmid-derived conjugation systems, indicating that conjugal transfer of lactococcal ICEs and plasmids involves genetically distinct machineries. Our genomic analysis and sequence-based classification of lactococcal ICEs creates a comprehensive overview of the conserved functional repertoires encoded by this family of MGEs in *L. lactis*, which can facilitate the future exploitation of the functional traits they encode by ICE mobilization to appropriate starter culture strains.

**Supplementary Information:**

The online version contains supplementary material available at 10.1186/s12864-024-10255-9.

## Introduction

The bacterium *Lactococcus lactis* is widely applied by the dairy industry for the fermentation of milk into products such as cheese, butter, and buttermilk. Besides the preservative effect of lactic acid production, *L. lactis* also plays a crucial role in the formation of flavor and texture characteristics of the fermented products. Mobile genetic elements (MGEs) like plasmids and integrative conjugative elements (ICEs) importantly contribute to the genomic diversity of the lactococci [1–3]. Plasmids are autonomously replicating extrachromosomal elements, and ICEs are integrated in the host chromosome where they are propagated passively during chromosomal replication and cell division. Representatives of both these MGEs have been reported to encode highly relevant industrial traits in *L. lactis*, like lactose, sucrose and α-galactoside utilization pathways, as well as the production of extracellular proteases, exopolysaccharides, and antimicrobial peptides (e.g., nisin) [4–8]. Both ICEs and conjugative plasmids can spread in *L. lactis* populations via conjugation, which requires cell-to-cell contact and the formation of a so-called mating pore through which the MGE material can be shared between the donor and recipient strain. The process of conjugation is similar for plasmids and ICEs, although ICEs have to excise from the chromosome to become conjugation competent. The machinery required for conjugation is commonly encoded entirely by the ICEs or conjugative plasmids itself, making these MGEs autonomously transferable. Contrary to the plasmid repertoire of *L. lactis* that has quite extensively studied [2], remarkably little is known about the ICEs in this species.

The ICE lifecycle can be summarized in several phases. Under normal conditions, ICEs remain dormant in the host chromosome with the conjugation genes remaining quiescent. When certain environmental conditions are encountered, or through stochastic gene-expression variation, the ICE genes involved in excision can become induced and the ICE excises from the host’s chromosome, representing the first stage of the ICE lifecycle. Most ICEs encode a tyrosine recombinase [9], which drives site-specific recombination of identical attachment sites (*att*) bordering the ICE. The excised ICE forms a circular double-stranded (ds) DNA molecule. Subsequently, the ICE genes encoding the conjugation machinery are expressed and their products are assembling to achieve mating pore formation (MPF), which accommodates conjugal transfer. A common mechanism of conjugative transfer is transfer via a type IV secretion system (T4SS) [10]. This T4SS is a multiple protein complex that spans the membrane and cell wall, in which a high degree of plasticity has been observed [11]. The only ubiquitous protein with homologs in all known T4SS is VirB4, an ATPase function that is essential for substrate transfer [12]. The circular, excised dsDNA ICE molecule is processed by the ICE-encoded relaxase that recognizes the origin of transfer sequence on the ICE (*oriT*), and nicks the dsDNA ICE to produce a linear, single stranded (ss) DNA-protein complex. This complex is recognized by the ICE-encoded coupling protein [13], to form the so-called transfer DNA (T-DNA). The conjugation machinery transfers the T-DNA across the mating pore and into the recipient cell. In the recipient cell, the ssDNA ICE re-circularizes and becomes double stranded through the activity of the recipient’s DNA polymerase, after which it recombines into the recipient’s chromosome. This repertoire of ICE-lifecycle associated functions is encoded by dedicated functional modules and operons by the ICE. Notably, modular exchanges of excision, conjugation and integration operons between ICEs have been reported to contribute to the overall diversity of this class of MGEs [9].

Analyses of bacterial genomes revealed that ICEs are wide-spread, and play a major role in the horizontal transfer of genes [14]. Eight distinguishable mating pore types have been described, two of which were reported to be specific for monoderm bacteria (e.g., gram-positives). Most of the ICEs found in monoderm bacteria encode the MPF_FA_ type mating pore [10], including the ICEs described in *L. lactis*.

Here, we present the results of automated ICE detection within *L. lactis* genomes. To this end we employed an automated Hidden Markov Model (HMM)-driven approach to not only identify genes that encode 5 of the core functions within the ICE lifecycle, but also assess their genetic linkage within the bacterial chromosome (co-localization), which is a prerequisite for their association with an ICE. This led us to identify 36 candidate ICEs in the genomes of 69 *L. lactis* strains, including both subspecies *cremoris* and subspecies *lactis*. In-depth analysis of the phylogenetic relatedness of 17 universally conserved-proteins of these lactococcal ICEs, classified them into 3 main ICE families and comparative analysis of the 17 shared functions underpinned their modular exchanges between ICEs of the same family as well as between ICE families. Finally, focusing on the conserved VirB4 ATPase function, we show that the ICE associated conjugation clusters are phylogenetically distinct from those found encoded by lactococcal conjugative plasmids, and indicate that the cross-species host-range of plasmid is broader than that of ICEs in *L. lactis*.

## Materials and methods

### Genomic data

The dataset used in this study encompasses *L. lactis* genomes available in the NCBI RefSeq database (version, May 2018) and represents 69 strains of this species (Supplemental table ST1), including both representatives of the subspecies *cremoris* and subspecies *lactis*. The delimited complete sequence of Tn*6098* was manually extracted from the *L. lactis KF147* genome assembly (Supplemental table ST1) using the defined *attp* sites reported in a previous study and used as an initial reference for all further phylogenetic analyses [4]. In addition, a homologue of Tn*5276* was extracted from the genome assembly of *L. lactis* CV56 (Supplemental table ST1) and was employed as a second well-characterized lactococcal ICE reference. This homologue was used since no complete genome was available for the strain in which Tn*5276* was originally identified (*L. lactis* R5 [15]). The sequences of the *L. lactis* plasmids that were used in this study were from a previously established database [3].

### Detection of ICEs and conjugative plasmids

The ICEs were found by using the *hmmsearch* function from HMMER v.3.2.1 [16], using the profile HMMS of the genes essential for the ICE lifecyle. We selected the following functions as for detection: integrase, N-acetylmuramidase, VirB4, relaxase and coupling protein. The corresponding PFAMs for these functions were selected from literature [14] and the HMMs found encoded by Tn*6098*. The PFAMs used for the search are listed in Table 1. Initial HMMer searches investigated whether each of the protein functions was encoded within individual *L. lactis* genome sequences, scoring for absence or presence using the manually curated positive-hit cut-off values provided by the PFAM database. Using the relaxase gene as a central gene, based on its location in both lactococcal ICE reference sequences (Tn*6098*, and Tn*5276*), we assessed whether the protein hits identified (at least one member of each PFAM) were co-localized within a 100 kbp genomic region of the lactococcal genome. Candidate ICEs were selected by detecting the colocalization (within 100 kbp) of minimally 4 of the 5 PFAMs elements, followed by manual inspection of the representation of the PFAMs and comparison of the candidate ICEs with the two reference ICE sequences.

**Table 1:**
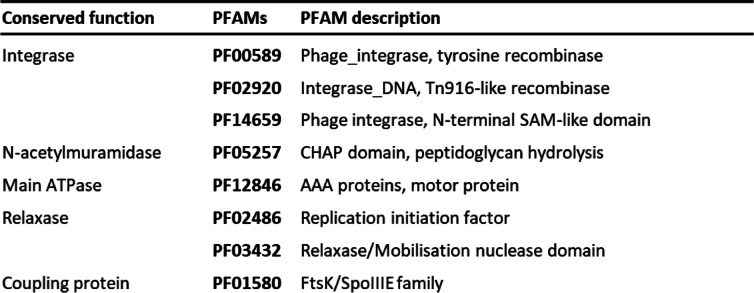
PFAM-HMM protein profiles used for the detection of conserved *L. lactis* ICE gene clusters

Plasmid-encoded VirB4 sequences were identified by the hmmsearch function using PFAM, PF12846 as query on a previously compiled database (2016) that encompassed 178 *L. lactis* derived plasmid sequences [3], yielding 27 positive hits. The 27 plasmid derived VirB4 sequences were manually curated, including the removal of duplicate entries and the requirement for full-length protein sequence alignment. The latter analysis was subsequently also used for HMM generation. Following these steps we ended up with 17 plasmid-derived, non-redundant VirB4 protein sequences.

### Identification of Lactococcal ICE core genes

Conservation of proteins encoded by the 36 candidate ICEs was assessed by BLASTP, using > 35% amino acid sequence similarity over > 80% of sequence length coverage as a cut-off. ICE associated core functions were defined as being present in at least 30 of the ICEs (~ 80% conserved). Arbitrary names were assigned to orthologous ICE-core proteins unless their function and protein name were proposed based on experimental evidence found in literature.

### HMM generation and verification

The amino acid sequences of the identified lactococcal ICE core proteins were aligned using the MAFFT (Multiple Alignment using Fast Fourier Transform, default settings) module available via the European Molecular Biology Laboratory (EMBL)-European Bioinformatics Institute (EBI). Phylogenetic trees were constructed from MAFFT alignments using the neighbor-joining tree without distance corrections method (EMBL-EBI; branch length: real) [17]. Protein clades were identified in the phylogenetic tree based on manual inspection of their phylogenetic clustering.

For each perceived protein-clade, a Hidden Markov Model (HMM) with the highest possible clade-discriminating capacity was generated by full-length alignment of the clade associated proteins using Multiple Sequence Comparison by Log- Expectation (MUSCLE) [18], and using the alignment output to generate clade-HMMs using the *hmmbuild* function in HMMER v3.2.1. All members of the ICE-core protein group were screened with the clade-derived HMMs using *hmmsearch*, and the clade discriminating capacity of the clade-derived HMMs was assessed by comparing their E-values. Provided that the poorest E-value within a clade was substantially better than the E-values obtained for protein members of the ICE-core protein group that were clustered into different clades, we consider the clade assignment confirmed.

### Phylogenetic tree generation

The phylogenetic trees used to illustrate the distribution of the plasmid and ICE derived VirB4 homologues were generated using the NCBI taxonomy browser. The bacterial representative refseq genomes were imported as a list and was rooted to contain all information. The resulting phylogenetic tree was exported in phylip tree format and imported to the iTol webserver [19]. Further image labeling was done using iTol.

### Gene map image generation

All gene map images were generated using EasyFig [20], and whole genbank comparison of encoded proteins (BLAST-P) were performed using the automated feature from Easyfig.

## Results

### 36 Lactococcal ICEs detected across 69 *L. lactis* genomes by searching for specific conserved ICE functions

In order to detect candidate ICEs in *L. lactis* genomes we employed a PFAM/HMM driven search engine that detects five of the established functions involved in specific stages of the ICE lifecycle. These five proteins were (i) the main ATPase VirB4 protein that is a constituent of the conjugative machinery required for ICE-transfer between cells, (ii) the muramidase that degrades the peptidoglycan during mate pair formation, (iii) the relaxase and (iv) coupling protein that play a vital role in T-DNA processing and cell-cell transfer, and finally (v) the tyrosine recombinase (integrase) that facilitates the mobilization in and out of a bacterial chromosome. Each of these functions is represented by HMMs present in the PFAM collection (Table 1), which were used to detect similar functions in *L. lactis* genomes encompassing both subspecies *cremoris* and *lactis* (Table 1). All of the 69 *L. lactis* genome dataset used here (Supplemental table ST1) that scored positive for at least 4 out of the 5 ICE-associated HMMs were taken along for further analysis. Since ICEs transfer as a single fragment it is expected that all ICE-associated functions co-localize in relative close proximity within the genome. This co-localization of the genes found in the *hmmer search* was assessed using the relaxase-encoding gene as a seed because it had the lowest frequency in the genomes among the targeted functions. Subsequently, we evaluated whether the other 3–4 functions were identified within 100 kbp from the relaxase-encoding gene. The positive hits fulfilling the co-localization criterion for at least 4 of the 5 features were flagged as a potential ICE, and selected for further manual investigation. During manual investigation the entries were compared with the two previously described model *L. lactis* ICEs (Tn*5276* and Tn*6098*). This method yielded a total of 36 putative ICEs across 69 lactococcal genomes (Supplemental table ST1). The majority of the ICEs are found in the subspecies *lactis* (27/45) and appeared less prevalent in the subspecies *cremoris* (2/24).

### The detected lactococcal ICEs are conserved in core gene composition but variable in length

**Fig. 1:**
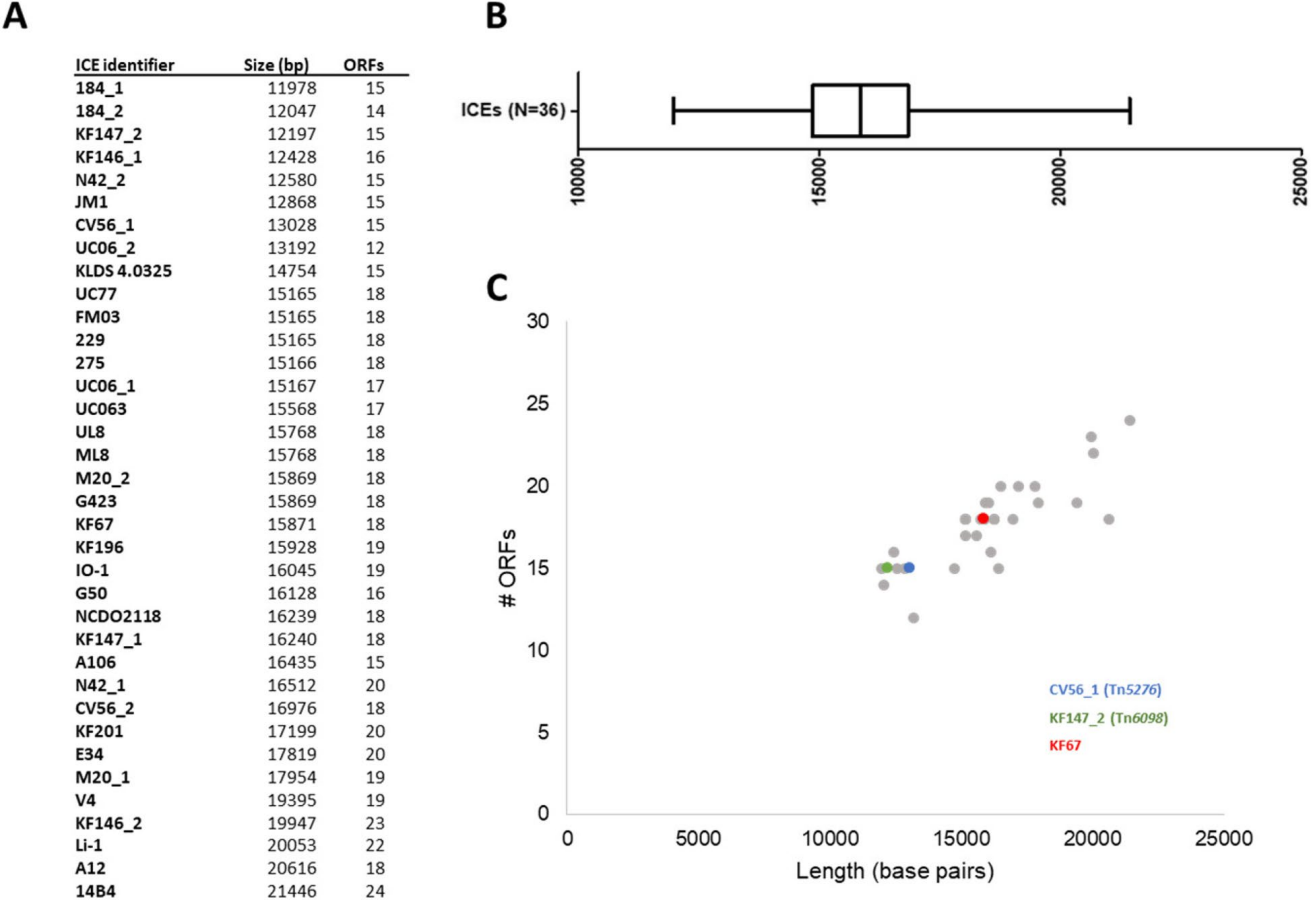
Distribution of variations found in the conserved ICE region. **A** Using the integrase and coupling protein as boundaries we determined the size in base pairs and the amount of ORFs for each of the detected ICEs. **B** A distribution box plot for all of the conserved regions. **C** The amount of ORFs plotted against the length (base pairs) with two reference ICE sequences and an example listed

Using the 36 candidate ICEs we investigated the positional conservation of the five identified core functions. Previous observations suggest that the integrase encoding gene is located close to the ICE boundary [9]. Consistently, we found the identified integrase genes always localized at one end of the five functions searched for, which we presume to be close to the ICE boundary or *att* site [9]. Further analysis identified the coupling protein encoding gene consistently located furthest from the integrase function, with the muramidase, VirB4 and relaxase encoding genes always in the same order between the integrase and coupling protein genes. These findings agree with the general conservation of the genetic structure of the core function clusters in ICEs. Nevertheless, the distance between the integrase and coupling protein varied between 12 and 21 kbp among the 36 candidate ICEs (Fig. 1A, B), which is in agreement with the number of ORFs found within these conserved ICE regions (Fig. 1C) and indicates a considerable degree of compositional variation of the candidate core-function regions of the lactococcal ICEs.

**Fig. 2:**
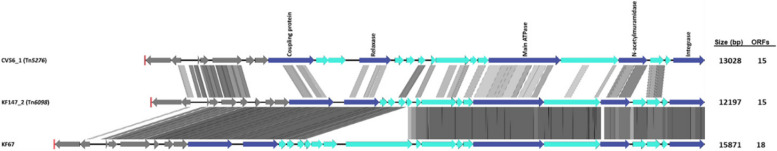
Conserved region comparison of three ICEs (CV56_1 (Tn*5276*), KF147_2 (Tn*6098*) and KF67). Aligned conserved ICE regions of three ICEs of varied size. In dark blue the five conserved ICE functions used for initial detection. The variation in size can be attributed to gene insertion / deletion between the conserved functions. The red bar indicates the cutoff used for the region definition, which was set to encompass all conserved core ICE genes as identified in our comparative analysis (see materials and methods for details)

To exemplify this compositional variation we aligned the core-function regions of three ICEs (Fig. 2), revealing a high overall similarity between the core-function regions of the candidate ICE identified in the genome of *L. lactis* KF67 in comparison to Tn*6098*, a 51 kb ICE that was previously identified in *L. lactis* KF147 [4]. Despite the observed core region synteny, the candidate ICE region of KF67 encompasses three additional genes inserted between the VirB4 and relaxase encoding genes, which encode two XRE-family transcriptional regulators and a cell surface protein of unknown function. Similar alignments also revealed that the core-function region of the Tn*5276*-resembling candidate ICE in the genome of *L. lactis* CV56 was more distant from Tn*6098* (Fig. 2), and was more similar to the partial information available for the original 70 kb Tn*5276* in strain R5 ([15]; data not shown). Nevertheless, the candidate ICE in strain CV56 shared several genes with Tn*6098* that were similarly positioned in both ICEs besides those used in the initial search, although interspaced with genes that were not found in Tn*5276*. These findings on the one hand establish that conservation of the ICE core function region exceeds beyond the five canonical functions we used to identify them, but also highlights that these lactococcal ICE’s display considerable variability in length and gene composition diversity in their core-function regions.

### Comparative analysis of conserved ICE functions

**Fig. 3:**
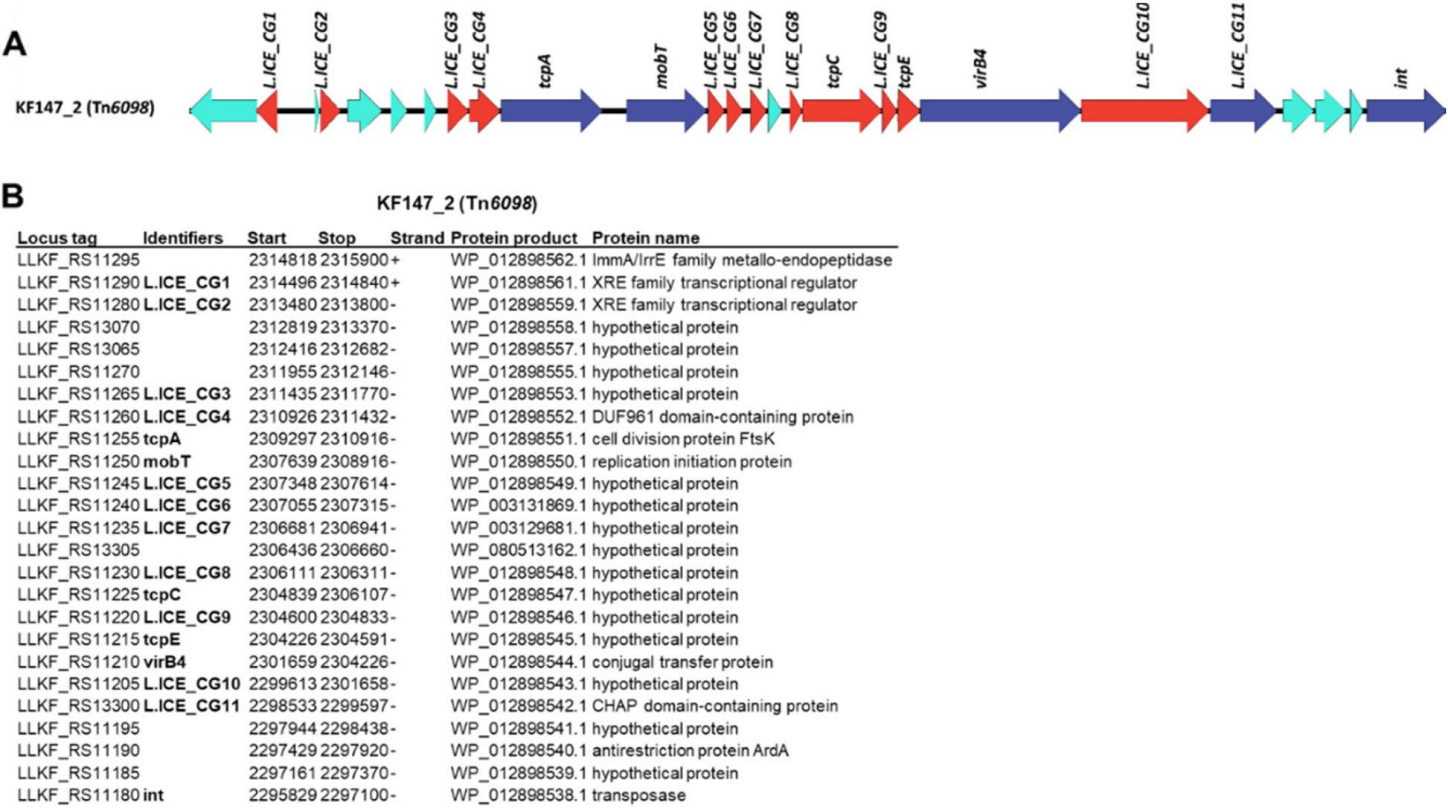
Example of the composition for the selected core lactococcal ICE genes (L.ICE_CGs). **A** In dark blue the 5 core ICE functions used for the initial detection, in red the core *L. lactis* ICE genes and in light blue the variable genes. **B** Gene table for KF147_2 and the assigned identifiers. The identifiers are taken from literature nomenclature of MPF_FA_ type systems, in order to allow future comparisons and easier access. The identifiers describe as followed: *tcpA =* coupling protein, *mobT =* relaxase, *tcpC* and *tcpE* two MPF associated genes, *virB4 =* ATPase function, *int* = integrase. The listed locus tags (first column) refer to the standardized locus tag numbering in the NCBI database, which differ from those in the original genome assembly, to facilitate their correspondence we provide the original locus tags for the first and last gene: LLKF_RS11295 = LLKF_2255, and LLKF_11180 = LLKF_2229

**Fig. 4:**
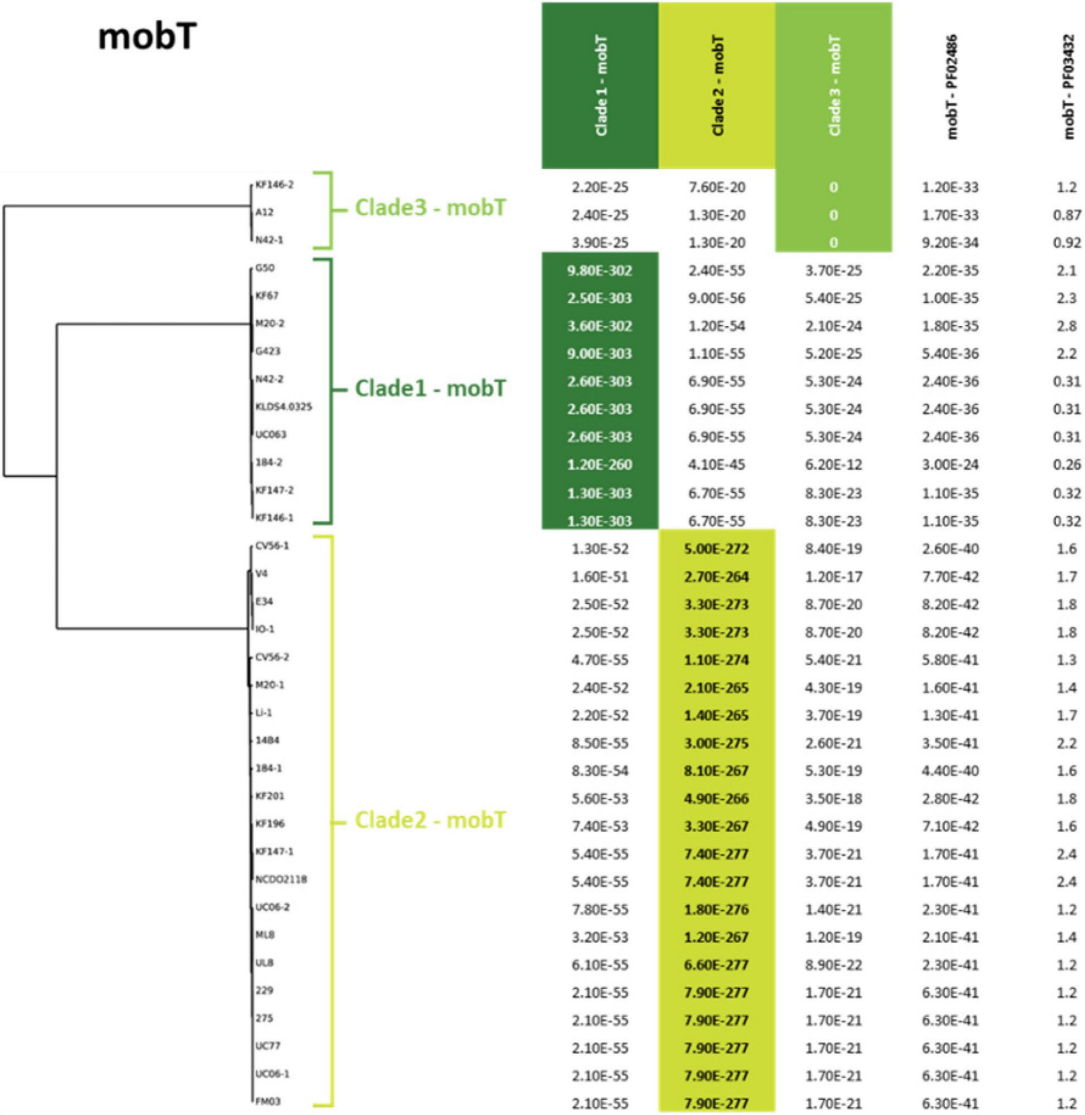
Example of the phylogenetic trees and clade definitions generated for each of the 17 core *L. lactis* ICE genes. In this example is the phylogenetic tree of the MobT function and the E-values of each clade specific HMM profile on each entry. A clear distribution and distinction of three clades can be discerned. Furthermore, the E-values are listed for the initial search for the ICE conserved features, illustrating that all lactococcal MobT proteins belong to the PF02486 protein family

**Fig. 5:**
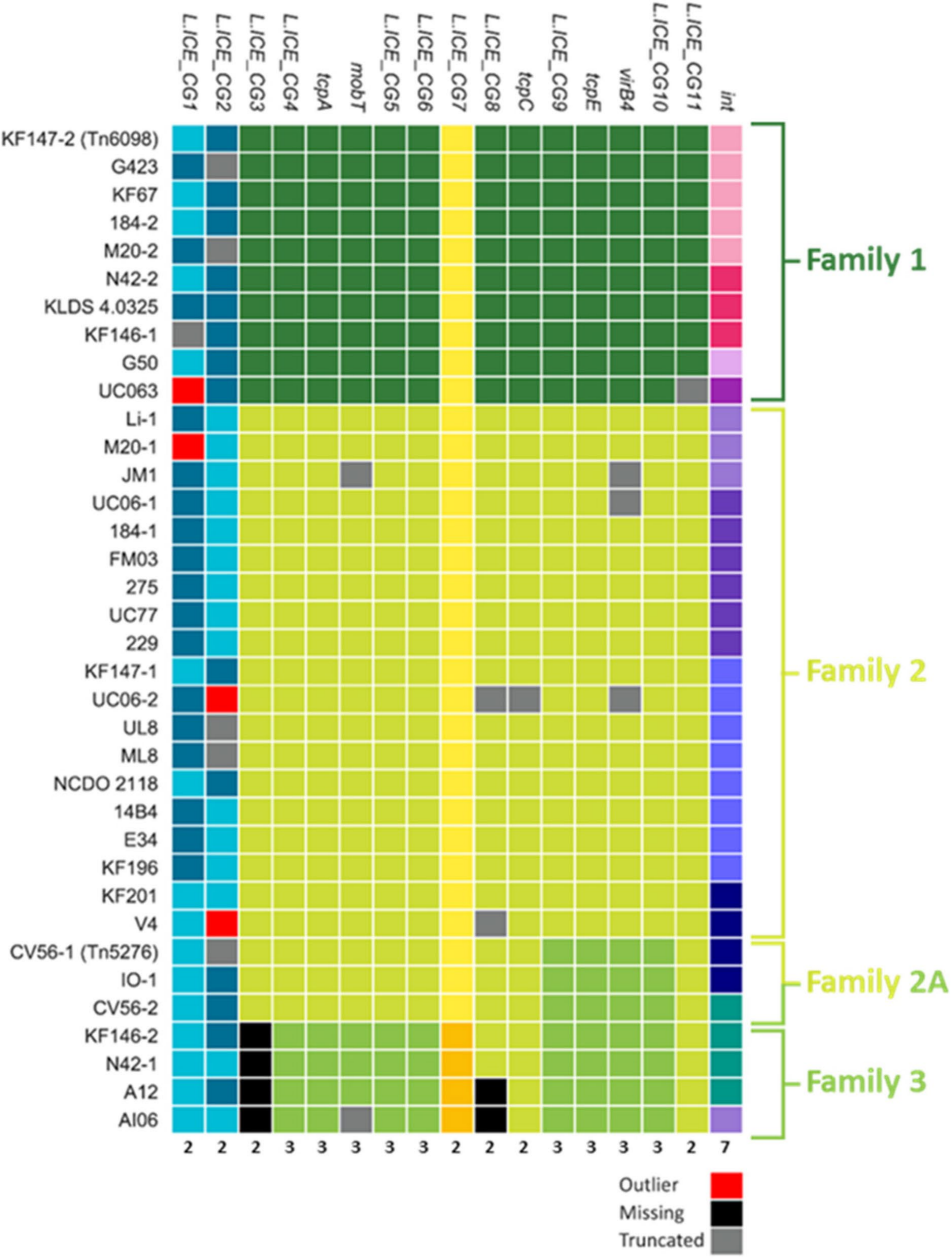
Heat map clustering of all identified *L. lactis* ICEs. Generated by compiling all the results of the clade definitions for each core *L. lactis* ICE gene. The colors correspond to different clades, and for exceptions (red for outliers, black for missing and grey for a truncation). By using the most obvious clustering (L.ICE_CG3 to CG6) the ICEs could be divided into three main families. Evidence of inter-family recombination is shown between family two and family three, by the interchange of at least the L.ICE_CG9 to CG10 functions. On the bottom of the heat map it is listed the total amount of clades in each L.ICE_CG function

Using Tn*6098*, we selected genes that are shared and conserved among the majority of the 36 detected candidate lactococcal ICEs (designated L.ICE_CGs). To this end, we identified all Tn*6098* ORFs that are present in at least 30 of the 36 ICEs, and exhibit at least 35% protein sequence identity in an alignment that covers at least 80% of the protein length. This analysis yielded 17 L.ICE_CGs, which besides the functions assigned to the initial five genes employed for ICE detection lack an assigned function in most cases (Fig. 3A and B). Notably, although the excisionase function (or Recombination Directional Factor [RDF], encoded by the *xis* gene) belongs to the essential ICE-mobilization functions and has been identified in Tn*6098* [4], the high sequence diversity of ICE-excisionases prevented its recognition among the L.ICE_CG functions and was therefore not taken along in the comparative analyses below. Multiple sequence alignment and phylogeny analysis of the conserved ICE-proteins was employed to identify distinct protein-sequence clades. When such sequence clades were identified, clade-specific HMMs were generated to facilitate the discrimination of these protein clades from the members of their L.ICE_CG group that were assigned to another clade. For example, the protein sequence alignment of the relaxase protein (MobT) of 34 lactococcal ICEs (2 truncated sequences derived from strains JM1 and AI06 were omitted) revealed three clearly distinct sequence clades that allowed the definition of clade-specific HMMs that allowed clade-specific classification of the MobT homologues (Fig. 4). Similar analyses were performed for each of the 17 conserved genes. The most obvious and coherent clade-assignments were detected for L.ICE_CG3, 4, 5, 6, TcpA, and MobT that in most cases coincided with congruent but less discriminant clade assignments of L.ICE_CG8, 9, 10, 11, TcpE and VirB4 (Fig. 5). These analyses indicated that based on clade classifications of these conserved functions, three ICE families can be readily recognized and that the majority of the detected candidate ICEs belonged to family 2 (19/36). Notably, three candidate ICEs displayed a mixed family assignment, i.e., the ICE regions identified in strains CV56–1, IO-1, and CV56–2 (Fig. 5). Out of the 36 candidate ICEs, 22 were detected as the only ICE present in a *L. lactis* strain, whereas in 7 strains two ICEs were detected that mostly classified in different ICE families (12/14), with a single exception in *L. lactis* CV56 that appeared to contain two ICEs assigned to family 2A. The modular character of the ICE core functions is clearly reflected by the observation that conserved functions that are predicted to be part of the conjugative machinery like L.ICE_CG9, *tcpE*, *virb4* and L.ICE_CG10 appear interchangeable in a collective manner (i.e., consistent clade-sets). This implies that these functions are not independently interchangeable, which is in agreement with the assumption that these proteins function in a multiprotein conjugation complex. Strikingly, the ICE family three consistently lacked a L.ICE_CG3 representative gene, while also L.ICE_CG8 appeared absent in 2 out of 4 representative ICEs of this family, while these functions were encoded by all members of ICE families 1 and 2 (Fig. 5). For L.ICE_CG1, CG2, and CG7 2 clades could be recognized, where the CG1 and CG2 clades did not clearly co-cluster with specific ICE families, while the distinguishable CG7 clades co-clustered with ICE families 1–2 and 3. Remarkably, the integrase (*int*) function displayed the highest degree of variation (Supplemental Figure SF2). The comparison of these integrases allowed the recognition of 7 clades of integrases that are represented by more than one member protein among the lactococcal ICEs. Notably, two of the integrase proteins could not be classified among these 7 (multimember) clades, implying that additional lactococcal integrase clades remain to be discovered, which were underrepresented in the genome collection used here (Supplemental Figure SF2). The integrases belonging to the 7 clades encompassing more than one member were dispersed over the different ICE families, although some ICE family enrichment of certain integrase clades could be recognized (Fig. 5). These findings imply that especially the boundaries of the conserved ICE region display a high degree of variation among ICE family members (i.e., lack of family-based clustering of CG1, 2 and *int*). Finally, five of the ICEs encode truncated proteins for at least one of the conserved ICE functions, which may interfere with conjugal transfer of these ICEs. For example, the truncations of the *virB4* gene in the ICEs encountered in strains JM1, UC06–1 and UC06–2 are highly likely to disable ICE transfer by these strains.

### Lactococcal ICEs VirB4 is distinct from the VirB4 of conjugative plasmids

**Fig. 6:**
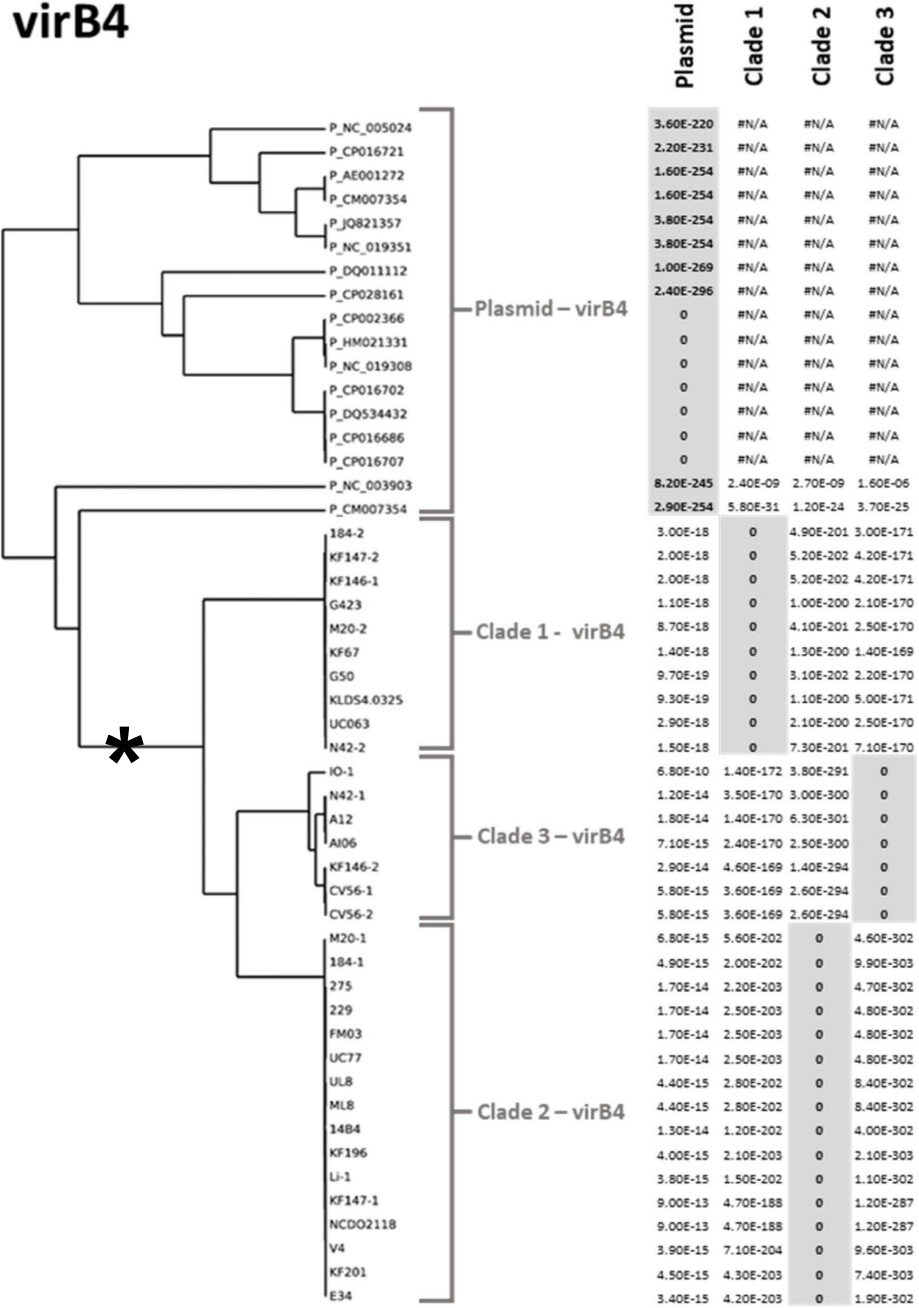
Phylogenetic tree and group definitions for VirB4s from both plasmids (P_) and ICEs. Each E-value is listed of each clade specific HMM profile on each entry. The plasmid derived VirB4 cluster definitely apart from the ICE derived VirB4s. Asterisk is the root of ICE derived VirB4 sequences

It has been proposed that MPF_T_ ICEs can undergo a lifestyle conversion into conjugative plasmids and vice versa [21]. In order to investigate whether the lactococcal MPF_FA_ ICEs also show indications of conversions into conjugative plasmids, we performed our conjugal cluster detection on available lactococcal plasmids (i.e., the plasmidome). To this end we employed the *virB4* encoded protein (targeted by the HMM PF12846) as the central target gene, since this protein has been shown to be conserved across all T4SS [12]. In the plasmid sequences used (see Materials and Methods for details), we identified 17 conjugative clusters. Although in some cases, some level of conservation of the order of conjugal-transfer associated genes could be recognized when comparing ICE and plasmid encoded gene-clusters, straightforward recognition of such synteny was hampered by the extensive sequence diversity observed between individual categories of gene functions in ICE and plasmid associated clusters (Supplemental Fig. SF1). In addition, in some plasmid associated conjugal transfer gene clusters a remarkably different sequence of encoded functions was encountered (Supplemental Fig. SF1). As anticipated, the conjugal plasmids did not encode an integrase function since the chromosomal integration is not part of the plasmid’s life cycle. From the 17 plasmid-borne conjugative clusters we selected the VirB4 amino acid sequences to compare these with their ICE orthologues. Protein sequence alignment revealed a clearly distinct clustering of plasmid and ICE derived VirB4 proteins (Fig. 6). In addition, this VirB4 protein sequence alignment allowed the definition of HMMs that robustly discriminate between plasmid and ICE derived VirB4 sequences. Within the plasmid VirB4 sequences two distinct clades appeared to be distinguishable that capture most of the plasmid VirB4 proteins, although two deviant sequences were classified as outliers (NC_003903 and CM007354). This finding suggests that distinct families might be recognized in the *L. lactis* associated conjugative plasmids using discriminative VirB4 HMMs.

### Lactococcal conjugal plasmids show a wider cross-species host range than the lactococcal ICEs

**Fig. 7:**
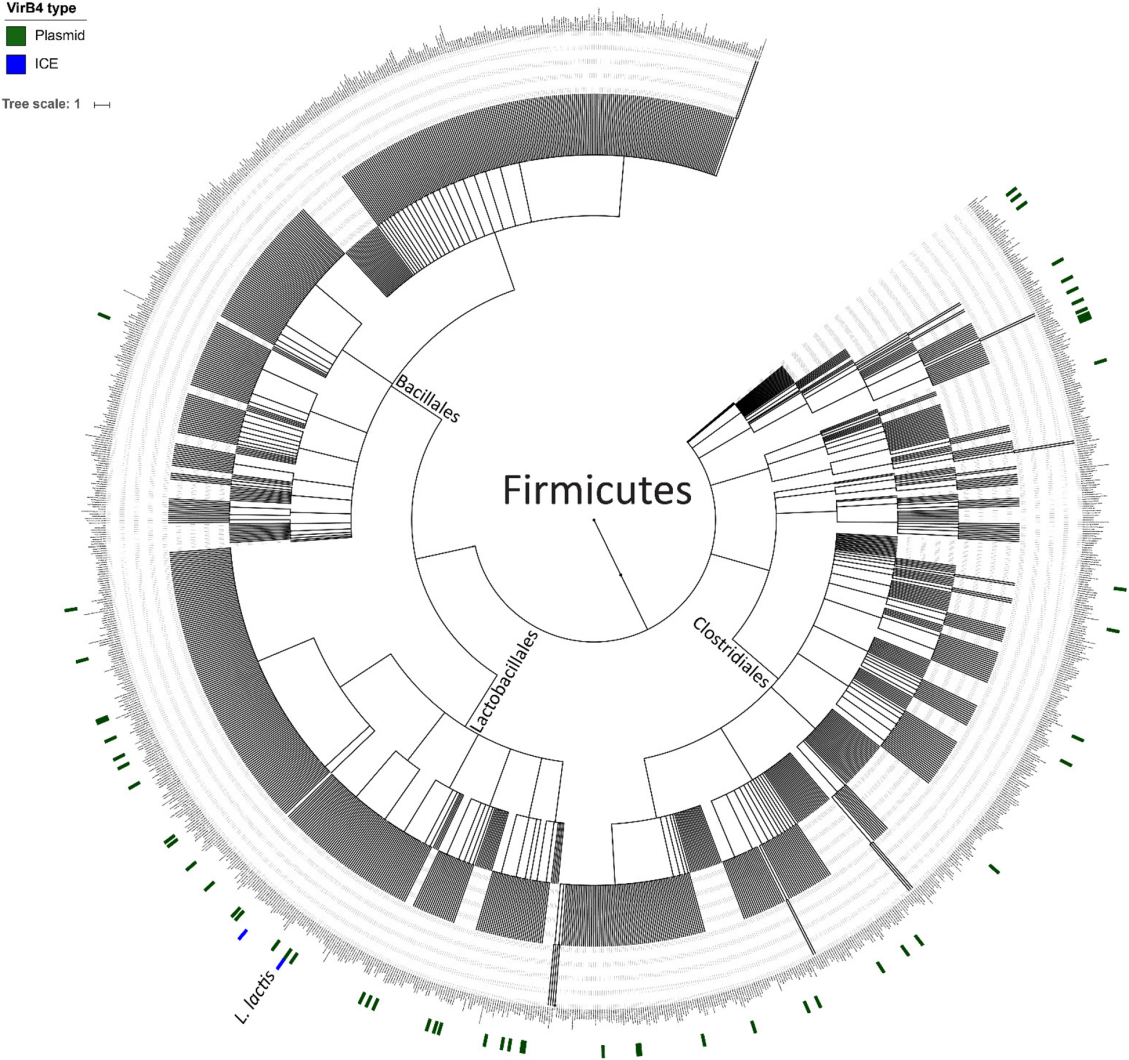
Mapping of the detected VirB4s on representative genomes of the Firmicutes. Both the ICE (blue) and plasmid (green) entries of *L. lactis* are depicted in the bottom. One additional ICE derived VirB4 could be detected, and was found within the Lactobacillales order. Of all the plasmid derived VirB4 entries found outside *L. lactis* the majority is represented within the Lactobacillales and Clostridiales orders, with only one representative found in the Bacillales order

It was previously proposed that MPF_T_ ICEs display a broader species host range as compared to the conjugative plasmids of the same type [21]. To investigate potential transfer events of either lactococcal plasmids or ICEs to other species, we performed an HMM search with the 4 VirB4 HMMs that could discriminate between the distinguishable ICE- and plasmid-derived VirB4 proteins using the prokaryotic representative genome set available at NCBI (5685 genomes). On basis of the poorest E-value obtained for the lactococcal reference sets, stringent HMM-score cutoffs were defined to avoid false-positive hits. This search yielded a list of 68 bacterial genomes that encompass either an ICE or plasmid VirB4 representative (Supplemental table ST2). Remarkably, the proteins identified were almost exclusively recognized (67 out of the 68) by HMM designed to recognize plasmid-derived lactococcal VirB4 proteins. The single ICE-derived VirB4 identified in a species other than *L. lactis* was found in a ruminant isolate of *Streptococcus entericus* DSM 14446. The positive hits obtained with the conjugative plasmid-derived VirB4 HMM were phylogenetically more widespread, with members of the phylum Firmicutes predominating (Fig. 7). The majority of these hits were found in the Lactobacillales and Clostridiales orders, with only a single hit in the Bacillales order. In addition, two hits were found outside the Firmicutes phylum, i.e., in representatives of the genera Streptomyces and Oligella (Supplemental figure SF3). These findings are in apparent contradiction with the proposed broader spread of ICE related conjugative gene clusters [21]. However, this previous search was conducted on MPF_T_ type conjugative elements of Gram-negative bacteria whereas in this study we looked at the MPF_FA_ type elements of the Gram-positive bacteria *L. lactis*.

## Discussion

In this study we investigated the prevalence of ICEs in the *L. lactis* species. For the initial ICE detection, we selected five conserved functions involved in the ICE lifecycle, and identified the available PFAM HMMs for each of these conserved functions. In order to minimize potential false negative search results, we included additional HMM designed for the detection of the integrase and relaxase functions. Using the relaxase function as a seed-function, we set a limit of 100.000 base pair as a region in which all five targeted functions should co-localize. Using this method, we identified 36 candidate ICEs in 69 lactococcal genomes. One of the strains that was analyzed in the present study (IO-1) was also investigated in a previous study that detected ICEs in 40 genomes of strains belonging to the Firmicutes [22]. Strikingly, this previous study reported the presence of two ICEs in this strain whereas our analysis only identified one ICE. This apparent discrepancy was found to be due to the stringency of our selection of the 4 out of 5 ICE core functions using the PFAM HMMs with their manually curated cut-offs. This caused the second ICE identified by Guédon and coworkers in *L. lactis* IO-1 to be excluded from our analysis because it presented only 3 of the 5 core function hits. The postulated VirB4 function in the second IO-1 ICE [22] actually has similarity scores with the PF12846 HMM that are substantially below the HMM-associated cut-off values, explaining why our analysis did not identify this possible second ICE in *L. lactis* IO-1.

To further investigate the diversity of these 36 ICEs we compiled a list of 17 genes shared amongst the majority of the ICEs (L.ICE_CGs) that not only share protein-sequence identity over their entire protein length, but their encoding genes are also localized at a similar position within the genetic context relative to the five functions used in the initial ICE detection (synteny). The phylogeny of the L.ICE_CGs was analyzed and led to the recognition of clades of sequences within these L.ICE_CGs that were distinguishable by clade-discriminating HMMs. Clade specification for the L.ICE-CGs allowed the classification of these identified candidate ICEs in *L. lactis* into three ICE families, which is strongly driven by the co-clustering of L.ICE_CG3-CG6 and L.ICE_CG8-CG11, although an exception to this co-clustering appeared to define an ICE subgroup within family 2. When comparing our family classifications to a previous study that assessed the diversity of ICEs in the genus *Streptococcus* [23], we conclude that all the lactococcal ICEs are members of the previously recognized Tn*916* superfamily. However, the refinement to ICE families in the analysis of streptococcal ICEs based on 2 VirB4 sequence clades (VirB4 1a and 1b [23];) was not feasible because the lactococcal VirB4 sequences clustered collectively separate from all the streptococcal VirB4 sequences, disallowing their co-classification in the previously proposed 1a and 1b subgroups [23]. In our analysis we propose not to use the VirB4 clades alone for the family classification of the ICEs in *L. lactis*, which would lead the family we designated as 2A to be grouped with family 3. Such family classification would ignore the separation based on the distinguishable L.ICE_CG7 function, which led us to propose the 3 family classification presented here. Taken together, the plasticity of the ICE-associated gene repertoire makes consistent definition of exact boundaries and criteria to define ICE families somewhat arbitrary due to their dependence on the genes and proteins that are taken along in the recognition of ICE families.

Our findings refine our phylogenetic knowledge of *L. lactis* ICEs, and corroborate that the evolution and diversification of ICEs is driven by the modular exchanges of genetic regions that encode homologous functions [24, 25]. Our results show that, although there is conservation in composition of the core ICE clusters, there is intergenic variation present between these L.ICE_CGs. This variation coincides with a remarkable size differences across the 36 ICEs. We separated the conserved clusters for each family, to investigate whether the size differences are conserved across the three main ICE-families (Supplemental figure SF4). Family 2 contains both the smallest and largest conserved regions and the inter-family variation in size underpins the plastic nature of ICEs. While ICEs can vary widely in size, ranging from 11.5 kbp (ICE in *S. aureus* USA300-FPR3757) [9] to 500 kbp (ICEMlSymR71 in *Mesorhizobium loti* R7A) [26]. The median size differs between distinct MPF types, with the ICEs of the MPF_FA_ type (to which the *L. lactis* ICEs belong) being reported to be the smallest (median of 23.5 kbp) [9]. Although in this study we looked at size comparisons of only the conserved ICE regions, encompassed between the integrase and coupling-protein encoding genes, we already found size variations ranging from 12 to 21 kbp. Since delimitation of the complete lactococcal ICEs (i.e., including the so-called cargo region of the ICE) was beyond the scope of this study, it remains to be determined what the average overall ICE-size is in *L. lactis*. The size variation in the core-region of the lactococcal ICEs corresponds to variable insertion of genes into the conserved gene-repertoire that is most likely linked to variations in conserved functions such as mate pair formation or T-DNA processing. In the absence of verification of their mobilization activity in vivo, it is unclear how these additional core-genes influences ICE functions, and although they are likely to play a role in the ICE lifecycle it cannot be excluded that they belong to the ICE-cargo. Similar ‘hotspots’ for novel DNA acquisition in ICEs has been observed previously and supports the overall genetic plasticity of this family of MGEs [25].

Similar to the majority of ICEs, the lactococcal ICEs consistently appeared to encompass an integrase belonging to the tyrosine recombinase family (PF00589). No serine recombinases were identified in any of the lactococcal ICEs, although these recombinases as well as the DDE recombinases are quite often found in ICEs from other species [9, 14, 24]. The majority of the assigned L.ICE_CGs encode hypothetical proteins (7 / 11) that lack a function prediction, but their high degree of conservation across the *L. lactis* ICEs implies that they serve essential functions in the ICE lifecycle. The L.ICE_CG8 to CG11 that neighbor VirB4 and are co-conserved across families, are most likely associated with the formation of the conjugation machinery. The L.ICE_CG3 to CG6 are co-conserved with the MobT and coupling protein and are therefore likely involved in T-DNA processing. Although L.ICE_CG3 and CG8 are absent in (a subset) of representatives of ICE-family three, it could be that these functions are performed by alternative proteins although we can’t exclude that these functions are not essential in family 3 ICEs. Intriguingly, both L.ICE_CG1 and 2 are annotated as XRE family transcriptional regulators, and similar proteins were shown to regulate ICE mobilization in three MPF_FA_ class ICEs, namely ICEbs1, ICESt3 and ICESt1 [27–29]. In ICEbs1 the autoregulatory transcriptional repressor (ImmR) prevents expression of the excisionase encoding *xis* gene as well as the downstream encoded conjugation machinery and DNA processing functions. Repression of mobilization of ICEst1 and ICEst3 is suggested to occur through a similar mechanism involving the transcriptional regulators arp1 and arp2 that also belong to the XRE-type regulators. These similarities warrant further investigation of the role of L.ICE_CG1 and CG2 in regulation of mobilization of ICEs in *L. lactis*.

The taxonomy of *L. lactis* is divided into two main subspecies (*lactis* and *cremoris*) of which the *cremoris* subspecies is predominantly found in dairy environments, while the *lactis* subspecies is isolated from a wider variety of sources, including plant and dairy environments and displays a higher level of genetic diversity [3]. Here we found that the *lactis* subspecies was overrepresented compared to the *cremoris* subspecies in the analysis of ICE presence. Adaptation to the dairy environment is associated with the loss of functions that are not providing a fitness benefit in the dairy niche [30–32], some of these functions could be encoded by readily removable ICEs. This notion is supported by the observed loss of ICE Tn*6098* in plant isolated *L. lactis* KF147 during its adaptation to growth in milk [33].

It has been proposed that ICEs and conjugative plasmids share a common ancestor, can exchange genetic modules, and even show an interchangeable lifestyle that blurs the distinction between these types of MGEs [21]. This proposition was largely based on the comparison of ICEs and conjugative plasmids of the MPF_T_ type that are found in Proteobacteria. Eight MPF (mating pair formation) types can be recognized based on VirB4 phylogenetic analysis, where the MPF_T_ type is one of the six types associated with diderms (i.e., bacteria with an outer membrane, typically Gram-negatives) that is the predominating MPF type in Proteobacteria [9, 10]. The MPF_FA_ type is one of the two MPF types recognized in monoderms (i.e., bacteria that lack an outer membrane, typically Gram-positives) that is found in Firmicutes and Actinobacteria [9, 10]. Here we conclude that at least for *L. lactis*, this interchangeability of ICE and conjugative-plasmid lifestyles is not observed for MPF_FA_ class ICEs, which is based on the complete lack of overlap of the sequence clades of the canonical VirB4 function between lactococcal conjugative plasmids and ICEs. Moreover, where previous analyses indicated that the ICEs of the MPF_T_ type had a broader host range compared to the conjugative plasmids of the same type, our analysis of the phylogenetic distribution of the lactococcal VirB4 clades indicates a broader host range of the lactococcal conjugative plasmids as compared to the lactococcal ICEs. Notably, our mechanistic understanding of the determinants of host range and phylogenetic distribution remains very limited, which warrants further molecular investigation of the observed differential ranges of phylogenetic width of the host range of MPF_T_ and MPF_FA_ type ICEs. The limited host-range of the MPF_FA_ type ICEs may be a feature specific for the lactococcal ICEs that is not shared by other species and genera [23] and extended and high resolution (like we performed here) in silico analyses of the phylogenetic width of conjugative plasmids and MPF_FA_ type ICEs (e.g., extended beyond VirB4 alone) in various Firmicutes species might provide a more detailed view on their core-function composition and their postulated lifestyle interchangeability.

In summary, our results create an inventory of the *L. lactis* ICEs and their phylogenetic relatedness, which enabled the distinction of three main families of ICEs and supports the role of modular genetic exchanges in the diversification of ICEs in this species. Moreover, our detailed analysis of VirB4 did not reveal any evidence for lifestyle interchangeability between ICEs and conjugative plasmids, suggesting that the common functions involved in mobilization of the MGEs have evolved separately in this species. The VirB4 analysis also indicated that the phylogenetic distribution of *L. lactis* ICEs appear to be species-restricted, whereas there is ample evidence for more extensive phylogenetic mobility of the lactococcal conjugative plasmids, which suggests a greater role for these plasmids in the genetic diversification of *L. lactis* in the context of the acquisition of novel functions from other species. Overall, this study increases our insight in the diversity of conjugative MGEs that play a key role in shaping the lactococcal genetic repertoire and thereby play a prominent role in the diversification of this species.

### Supplementary Information

**Supplementary Material 1.**

## Data Availability

The NCBI accession numbers for the genome assemblies used in this study are listed in Table ST1.
