# Supplementary material for: Detection and classification of the integrative conjugative elements of Lactococcus lactis
Source: BMC Genomics. 2024 Apr 1;25:324. doi: 10.1186/s12864-024-10255-9 (PMC10983677; doi:10.1186/s12864-024-10255-9)
Supplement: Supplementary file 1 — Supplementary Material 1. [file 12864_2024_10255_MOESM1_ESM.pdf]

Supplementary Tables and Figures for:

Detection and classification of the integrative conjugative elements of *Lactococcus lactis*

Simon van der Els[a,b,c], Reshtrie Sheombarsing[a,b], Thijn van Kempen[b], Michiel Wels[b], Jos Boekhorst[a], Peter A. Bron[b,c], and Michiel Kleerebezem[a,c]

[a] Host-Microbe Interactomics Group, Department of Animal Sciences, Wageningen University & Research, De Elst 1, 6708 WD, Wageningen, The Netherlands.

[b] NIZO B.V., Kernhemseweg 2, 6718 ZB, Ede, The Netherlands.

[c] BE-Basic Foundation, Mijnbouwstraat 120, 2628 RX, Delft, The Netherlands.

Supplemental table ST1. Strains and genomes, and number of ICEs found in this study

(sub-) species		strain	Isolation source	N° contigs	size (Mbp)	NCBI-Assembly	N° ICEs
Lactococcus	*cremoris*	158	Dairy fermentation	8	2.5	GCA_002078375.2	0
Lactococcus	*cremoris*	3107	Dairy	7	2.5	GCA_003394085.1	0
Lactococcus	*cremoris*	A76	Cheese production	5	2.6	GCA_000236475.1	0
Lactococcus	*cremoris*	AM2	Dairy starter	255	2.5	GCA_001622405.1	0
Lactococcus	*cremoris*	B40	Dairy starter	9	2.8	GCA_030386275.1	0
Lactococcus	*cremoris*	C4	Yogurt	5	2.6	GCA_003966935.1	0
Lactococcus	*cremoris*	FG2	Dairy starter	374	2.6	GCA_001622365.1	0
Lactococcus	*cremoris*	HP	Dairy starter	311	2.4	GCA_001622215.1	0
Lactococcus	*cremoris*	JM1	Dairy fermentation	8	2.8	GCA_002078895.1	1
Lactococcus	*cremoris*	JM2	Dairy fermentation	5	2.6	GCA_002078915.1	0
Lactococcus	*cremoris*	JM3	Dairy fermentation	6	2.6	GCA_002078935.1	0
Lactococcus	*cremoris*	JM4	Dairy fermentation	6	2.5	GCA_002078955.1	0
Lactococcus	*cremoris*	KW10	Kaanga Wai	46	2.4	GCA_001622205.1	0
Lactococcus	*cremoris*	KW2	NA	1	2.4	GCA_000468955.1	0
Lactococcus	*cremoris*	LMG6897	Cheese starter	271	2.4	GCA_001622295.1	0
Lactococcus	*cremoris*	MG1363	NA	1	2.5	GCA_000009425.1	0
Lactococcus	*cremoris*	N41	Soil and grass	182	2.6	GCA_001622285.1	0
Lactococcus	*cremoris*	NCDO763	Dairy starter	156	2.5	GCA_001622385.1	0
Lactococcus	*cremoris*	NZ9000	NA	1	2.5	GCA_000143205.1	0
Lactococcus	*cremoris*	SK11	NA	6	2.6	GCA_000014545.1	0
Lactococcus	*cremoris*	SK110	Dairy starter	212	2.5	GCA_001622375.1	0
Lactococcus	*cremoris*	UC109	Dairy fermentation	8	2.4	GCA_002078765.2	0
Lactococcus	*cremoris*	UC509.9	NA	9	2.5	GCA_000312685.1	0
Lactococcus	*cremoris*	V4	Raw sheep milk	2	2.6	GCA_032586255.1	1
Lactococcus	*lactis*	184	NA	113	2.6	GCA_009661795.1	2
Lactococcus	*lactis*	229	Dairy fermentation	6	2.6	GCA_002078415.1	1
Lactococcus	*lactis*	275	Dairy fermentation	5	2.8	GCA_002078435.1	1
Lactococcus	*lactis*	14B4	Almond drupe	2	2.6	GCA_003176835.1	1
Lactococcus	*lactis*	A12	NA	5	2.7	GCA_900088425.1	1
Lactococcus	*lactis*	AI06	Mesocarp	1	2.4	GCA_000761115.1	1
Lactococcus	*lactis*	ATCC19435	Dairy starter	171	2.5	GCA_001456385.1	0
Lactococcus	*lactis*	CV56	Vagina of healthy women	6	2.5	GCA_000192705.1	2
Lactococcus	*lactis*	DRA4	Dairy starter	253	2.5	GCA_001622235.1	0
Lactococcus	*lactis*	E34	Silage	46	2.4	GCA_001456455.1	1
Lactococcus	*lactis*	F44	Medium	1	2.4	GCA_002804185.1	0
Lactococcus	*lactis*	FM03	10 weeks old Samso 45+ cheese	8	2.5	GCA_002148215.1	1
Lactococcus	*lactis*	G423	Medium	1	2.4	GCA_002804285.1	1
Lactococcus	*lactis*	G50	Napier grass	1	2.3	GCA_002895225.1	1
Lactococcus	*lactis*	IL1403	NA	1	2.4	GCA_003722275.1	0
Lactococcus	*lactis*	IL6288	NA	1	2.2	GCA_003722255.1	0
Lactococcus	*lactis*	IO-1	NA	1	2.4	GCA_000344575.1	1
Lactococcus	*lactis*	K231	White kimchii	3	2.4	GCA_032464685.1	0
Lactococcus	*lactis*	K337	White kimchii	61	2.4	GCA_001456525.1	0
Lactococcus	*lactis*	KF134	Alfalfa and radish sprouts	33	2.5	GCA_001456725.1	0
Lactococcus	*lactis*	KF146	Alfalfa and radish sprouts	2	2.6	GCA_032464735.1	2
Lactococcus	*lactis*	KF147	Sprouts	2	2.6	GCA_000025045.1	2
Lactococcus	*lactis*	KF196	Japanese kaiwere shoots	38	2.4	GCA_001456595.1	1
Lactococcus	*lactis*	KF201	Sliced mixed vegetables	62	2.4	GCA_001456755.1	1
Lactococcus	*lactis*	KF24	Alfalfa sprouts	192	2.6	GCA_001456505.1	0
Lactococcus	*lactis*	KF282	Mustard and cress	98	2.7	GCA_001456615.1	0
Lactococcus	*lactis*	KF67	Grapefruit juice	110	2.7	GCA_001456545.1	1
Lactococcus	*lactis*	KF7	Alfalfa sprouts	2	2.4	GCA_032464755.1	0
Lactococcus	*lactis*	KLDS4.0325	Home-made koumiss	7	2.8	GCA_000479375.3	1
Lactococcus	*lactis*	Li-1	Grass	75	2.5	GCA_001456775.1	1
Lactococcus	*lactis*	LMG14418	Bovine milk	225	2.4	GCA_001456695.1	0
Lactococcus	*lactis*	LMG8526	Chinese radish seeds	4	2.5	GCA_032464775.1	0
Lactococcus	*lactis*	LMG9446	Frozen peas	133	2.5	GCA_001456675.1	0
Lactococcus	*lactis*	LMG9447	Frozen peas	227	2.7	GCA_001456685.1	0
Lactococcus	*lactis*	M20	Soil	149	2.7	GCA_001456785.1	2
Lactococcus	*lactis*	ML8	Dairy starter	4	2.7	GCA_032464785.1	1
Lactococcus	*lactis*	N42	Soil and grass	6	2.9	GCA_032464835.1	2
Lactococcus	*lactis*	NCDO2118	Frozen peas	2	2.6	GCA_000478255.1	1
Lactococcus	*lactis*	NCDO895	Dairy starter	198	2.5	GCA_001456865.1	0
Lactococcus	*lactis*	S0	Fresh raw milk	1	2.5	GCA_000807375.1	0
Lactococcus	*lactis*	UC06	Dairy fermentation	4	2.7	GCA_002078975.1	2
Lactococcus	*lactis*	UC063	Dairy fermentation	6	2.5	GCA_002078495.1	1
Lactococcus	*lactis*	UC317	Dairy starter	213	2.5	GCA_001456855.1	0
Lactococcus	*lactis*	UC77	Dairy fermentation	3	2.6	GCA_002078615.1	1
Lactococcus	*lactis*	UL8	Dairy fermentation	4	2.5	GCA_002078855.1	1

Supplemental table ST2. List of genomes containing a plasmid or ICE VirB4 representative.

	Bacterium	Plasmid Value	ICE-group 1 Value	ICE-group 2 Value	ICE-group 3 Value
A3GW_RS0109490	Streptococcus entericus DSM 14446	3.50E-16	6.80E-198	2.90E-224	3.50E-194
BS638_RS05430	Clostridium tepidum	2.90E-254	5.80E-31	1.20E-24	3.70E-25
HMPREF0072_RS02720	Anaerococcus lactolyticus ATCC 51172	2.90E-254	5.80E-31	1.20E-24	3.70E-25
BUB96_RS09725	Atopostipes suicloacalis DSM 15692	2.90E-254	5.80E-31	1.20E-24	3.70E-25
BQ7376_RS04275	Massiliomicrobiota timonensis	2.90E-254	5.80E-31	1.20E-24	3.70E-25
IO99_RS16110	Clostridium sulfidigenes	2.90E-254	5.80E-31	1.20E-24	3.70E-25
AWM71_RS07100	Aerococcus christensenii	2.90E-254	5.80E-31	1.20E-24	3.70E-25
RH95_RS06950	Peptoniphilus rhinitidis 1-13	2.90E-254	5.80E-31	1.20E-24	3.70E-25
BQ4451_RS08475	Anaerococcus mediterraneensis	2.90E-254	5.80E-31	1.20E-24	3.70E-25
BUA73_RS08150	Hathewaya proteolytica DSM 3090	2.90E-254	5.80E-31	1.20E-24	3.70E-25
PTSHG_RS01970	Peptoniphilus senegalensis JC140	2.90E-254	5.80E-31	1.20E-24	3.70E-25
F594_RS0101685	Peptoniphilus lacrimalis DSM 7455	2.90E-254	5.80E-31	1.20E-24	3.70E-25
BN6539_RS16730	Emergencia timonensis	2.90E-254	5.80E-31	1.20E-24	3.70E-25
B092_RS0107970	Oligella ureolytica DSM 18253	2.90E-254	5.80E-31	1.20E-24	3.70E-25
DO83_RS14900	Anaerostipes hadrus	2.90E-254	5.80E-31	1.20E-24	3.70E-25
I872_RS07915	Streptococcus cristatus AS 1.3089 (Streptococcus oligofermentans AS 1.3089)	2.90E-254	5.80E-31	1.20E-24	3.70E-25
SANR_RS05535	Streptococcus anginosus C238	2.90E-254	5.80E-31	1.20E-24	3.70E-25
CLCOL_RS03885	Clostridium colicanis DSM 13634	2.90E-254	5.80E-31	1.20E-24	3.70E-25
STRPS_RS01625	Streptococcus pseudoporcinus LQ 940-04	2.90E-254	5.80E-31	1.20E-24	3.70E-25
BN30_RS02155	Fenollaria massiliensis	2.90E-254	5.80E-31	1.20E-24	3.70E-25
SPSINT_RS10770	Staphylococcus pseudintermedius HKU10-03	2.90E-254	5.80E-31	1.20E-24	3.70E-25
BUA48_RS05235	Clostridium collagenovorans DSM 3089	2.90E-254	5.80E-31	1.20E-24	3.70E-25
HMPREF0351_12743	Enterococcus faecium DO	2.90E-254	5.80E-31	1.20E-24	3.70E-25
SSUBM407_0487	Streptococcus suis BM407	2.90E-254	5.80E-31	1.20E-24	3.70E-25
SSUBM407_0977	Streptococcus suis BM407	2.90E-254	5.80E-31	1.20E-24	3.70E-25
C231_RS0104905	Allofustis seminis DSM 15817	8.40E-254	2.20E-31	4.10E-25	1.20E-25
HMPREF9707_RS03280	Facklamia ignava CCUG 37419	8.90E-254	7.80E-31	6.10E-24	1.90E-24
HMPREF9706_RS05570	Facklamia hominis CCUG 36813	9.20E-254	6.10E-31	2.40E-24	4.70E-25
RA45_RS06060	Enterococcus cecorum	1.00E-251	2.90E-31	1.10E-23	4.10E-25
BN162_RS06940	Holdemania massiliensis AP2	1.70E-250	1.40E-29	3.20E-25	6.80E-25
DAHG_RS12270	Dielma fastidiosa	1.70E-250	1.40E-29	3.20E-25	6.80E-25
HMPREF9286_RS07560	Peptoniphilus harei ACS-146-V-Sch2b	2.10E-249	4.00E-30	1.20E-25	2.40E-25
CD630_05020	Clostridioides difficile 630 (Clostridium difficile 630)	3.40E-248	1.50E-29	1.60E-24	1.20E-23
HMPREF9083_RS05410	Dialister micraerophilus DSM 19965	4.50E-248	6.20E-30	1.70E-26	2.40E-25
HMPREF9225_RS07390	Peptoniphilus duerdenii ATCC BAA-1640	4.50E-248	6.20E-30	1.70E-26	2.40E-25
BQ7400_RS05170	Urinacoccus massiliensis	4.50E-248	6.20E-30	1.70E-26	2.40E-25
HMPREF0389_RS05525	Filifactor alocis ATCC 35896	4.50E-248	6.20E-30	1.70E-26	2.40E-25
BM057_RS09090	Pisciglobus halotolerans	2.20E-246	9.60E-30	1.00E-25	1.50E-25
H541_RS0104015	Bavariicoccus seileri DSM 19936	5.80E-246	1.30E-28	2.50E-26	7.50E-26
SCP1.146	Streptomyces coelicolor A3(2)	8.20E-245	2.40E-09	2.70E-09	1.60E-06
C270_RS08540	Leuconostoc carnosum JB16	1.70E-240	#N/A	#N/A	#N/A
OKIT_RS04405	Oenococcus kitaharae DSM 17330	1.80E-240	#N/A	#N/A	#N/A
PECL_RS09215	Pediococcus claussenii ATCC BAA-344	4.70E-240	#N/A	#N/A	#N/A
FD50_RS00495	Lactobacillus satsumensis DSM 16230 = JCM 12392	1.30E-239	#N/A	#N/A	#N/A
IV55_RS08695	Lactobacillus siliginis	1.30E-238	#N/A	#N/A	#N/A
LOOC260_RS11580	Lactobacillus hokkaidonensis JCM 18461	1.80E-238	#N/A	#N/A	#N/A
FC85_RS00310	Lactobacillus diolivorans DSM 14421	3.10E-238	#N/A	#N/A	#N/A
IWT5_RS11015	Lactobacillus mixtipabuli	3.80E-238	#N/A	#N/A	#N/A
FC82_RS08580	Lactobacillus collinoides DSM 20515 = JCM 1123	3.90E-238	#N/A	#N/A	#N/A
FC67_RS04030	Lactobacillus alimentarius DSM 20249	7.00E-238	#N/A	#N/A	#N/A
OKIT_RS02580	Oenococcus kitaharae DSM 17330	8.40E-238	#N/A	#N/A	#N/A
WANG_RS11285	Lactobacillus kefiranofaciens ZW3	8.90E-238	#N/A	#N/A	#N/A
LSE01S_RS10185	Lactobacillus senmaizukei DSM 21775 = NBRC 103853	4.10E-237	#N/A	#N/A	#N/A
BUB96_RS09320	Atopostipes suicloacalis DSM 15692	6.10E-237	4.90E-27	3.60E-25	2.90E-24
WOSG25_RS06670	Lactobacillus oryzae JCM 18671	6.80E-237	#N/A	#N/A	#N/A
pWCFS103_32	Lactobacillus plantarum WCFS1	5.40E-235	#N/A	#N/A	#N/A
ARB84_RS06575	Fusicatenibacter saccharivorans	5.20E-226	1.60E-28	2.10E-26	8.00E-25
GCWU000342_RS08470	Shuttleworthia satelles DSM 14600	2.00E-225	6.50E-31	6.30E-27	2.20E-28
CD630_33380	Clostridioides difficile 630 (Clostridium difficile 630)	2.10E-225	1.10E-28	1.90E-26	7.80E-25
BN3267_RS12355	Fournierella massiliensis	2.70E-225	2.10E-28	1.30E-26	9.20E-25
Q783_RS11595	Carnobacterium inhibens subsp. gilichinskyi	5.00E-225	#N/A	#N/A	#N/A
Q783_RS11380	Carnobacterium inhibens subsp. gilichinskyi	5.20E-225	#N/A	#N/A	#N/A
BLT48_RS08560	Carnobacterium viridans	7.20E-225	#N/A	#N/A	#N/A
HMPREF1085_RS13625	[Clostridium] bolteae 90A9	1.30E-224	1.80E-28	3.80E-26	4.20E-24
FC89_RS02720	Lactobacillus ghanensis DSM 18630	3.30E-224	#N/A	#N/A	#N/A
RUMOBE_RS13330	Blautia obeum ATCC 29174	3.90E-224	1.00E-28	2.00E-27	1.30E-24
BLT48_RS00355	Carnobacterium viridans	1.40E-223	#N/A	#N/A	#N/A
BLT48_RS00930	Carnobacterium viridans	1.40E-223	#N/A	#N/A	#N/A

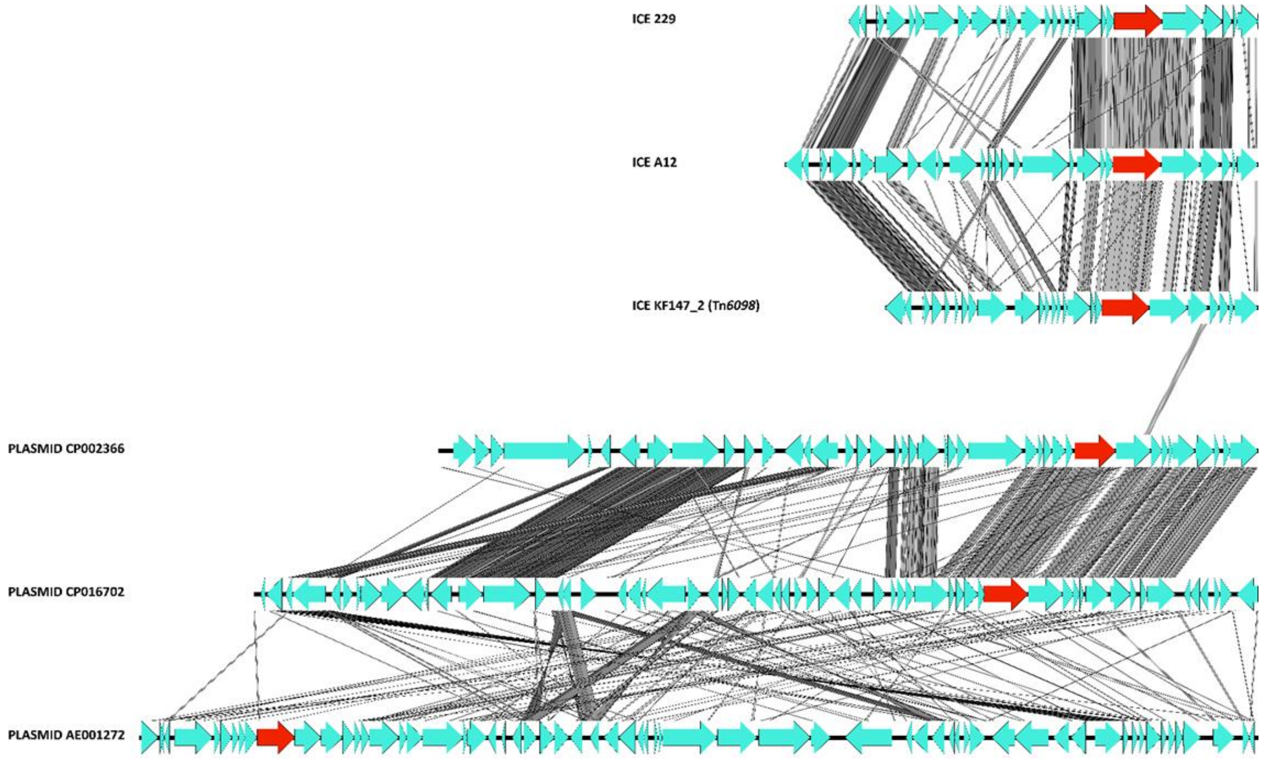

Supplemental figure SF1. Exemplary ICE conserved regions of the three main families compared to three *L. lactis* conjugal plasmids. Within the respective MGEs (ICEs and plasmids) there is conservation of structure and sequence (Blast scores), however between there is no high conservation observed. VirB4 is indicated in bright red.

int

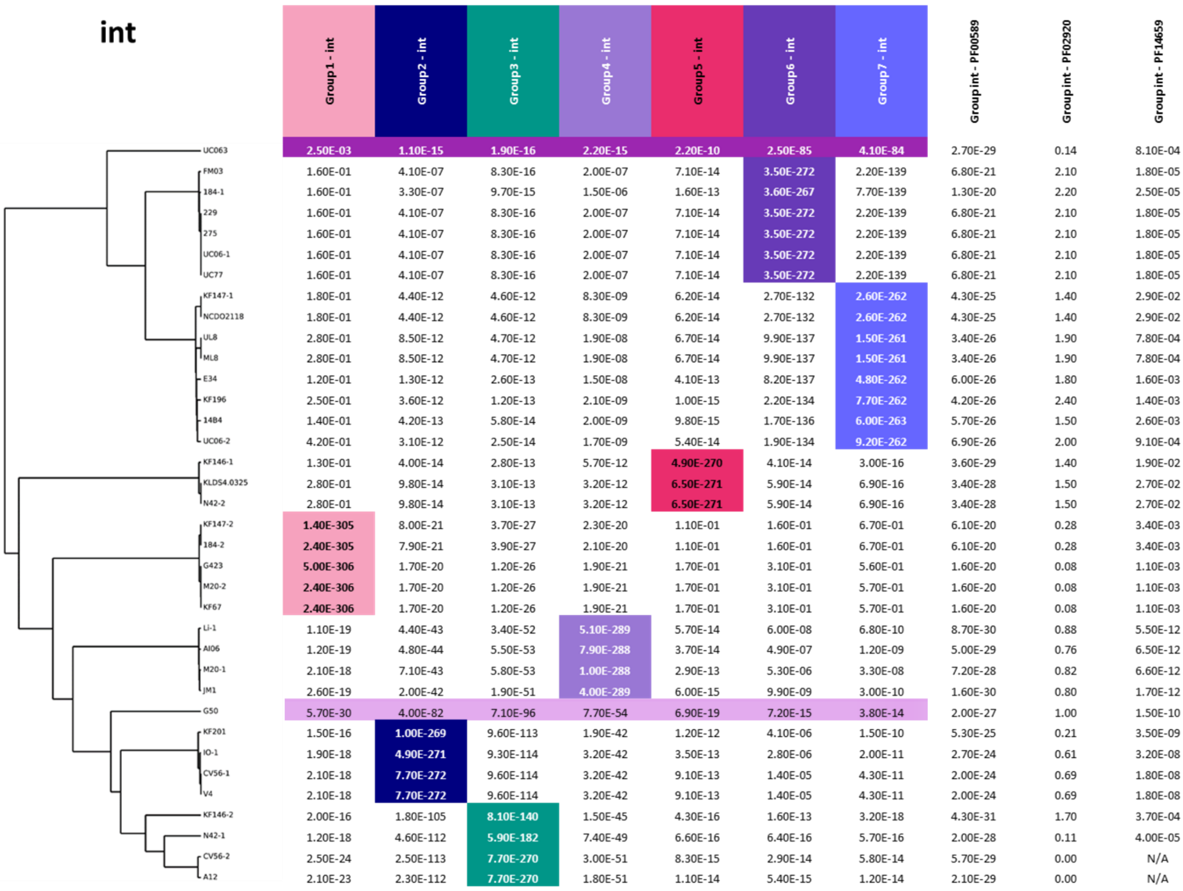

	Group1 - int	Group2 - int	Group3 - int	Group4 - int	Group5 - int	Group6 - int	Group7 - int	Group int - PF00589	Group int - PF02920	Group int - PF14659
UC063	2.50E-03	1.10E-15	1.90E-16	2.20E-15	2.20E-10	2.50E-85	4.10E-84	2.70E-29	0.14	8.10E-04
FM03	1.60E-01	4.10E-07	8.30E-16	2.00E-07	7.10E-14	3.50E-272	2.20E-139	6.80E-21	2.10	1.80E-05
184-1	1.60E-01	3.30E-07	9.70E-15	1.50E-06	1.60E-13	3.60E-267	7.70E-139	1.30E-20	2.20	2.50E-05
229	1.60E-01	4.10E-07	8.30E-16	2.00E-07	7.10E-14	3.50E-272	2.20E-139	6.80E-21	2.10	1.80E-05
275	1.60E-01	4.10E-07	8.30E-16	2.00E-07	7.10E-14	3.50E-272	2.20E-139	6.80E-21	2.10	1.80E-05
UC06-1	1.60E-01	4.10E-07	8.30E-16	2.00E-07	7.10E-14	3.50E-272	2.20E-139	6.80E-21	2.10	1.80E-05
UC77	1.60E-01	4.10E-07	8.30E-16	2.00E-07	7.10E-14	3.50E-272	2.20E-139	6.80E-21	2.10	1.80E-05
KF147-1	1.80E-01	4.40E-12	4.60E-12	8.30E-09	6.20E-14	2.70E-132	2.60E-262	4.30E-25	1.40	2.90E-02
NCDO2118	1.80E-01	4.40E-12	4.60E-12	8.30E-09	6.20E-14	2.70E-132	2.60E-262	4.30E-25	1.40	2.90E-02
UL8	2.80E-01	8.50E-12	4.70E-12	1.90E-08	6.70E-14	9.90E-137	1.50E-261	3.40E-26	1.90	7.80E-04
ML8	2.80E-01	8.50E-12	4.70E-12	1.90E-08	6.70E-14	9.90E-137	1.50E-261	3.40E-26	1.90	7.80E-04
E34	1.20E-01	1.30E-12	2.60E-13	1.50E-08	4.10E-13	8.20E-137	4.80E-262	6.00E-26	1.80	1.60E-03
KF196	2.50E-01	3.60E-12	1.20E-13	2.10E-09	1.00E-15	2.20E-134	7.70E-262	4.20E-26	2.40	1.40E-03
14B4	1.40E-01	4.20E-13	5.80E-14	2.00E-09	9.80E-15	1.70E-136	6.00E-263	5.70E-26	1.50	2.60E-03
UC06-2	4.20E-01	3.10E-12	2.50E-14	1.70E-09	5.40E-14	1.90E-134	9.20E-262	6.90E-26	2.00	9.10E-04
KF146-1	1.30E-01	4.00E-14	2.80E-13	5.70E-12	4.90E-270	4.10E-14	3.00E-16	3.60E-29	1.40	1.90E-02
KLDS4.0325	2.80E-01	9.80E-14	3.10E-13	3.20E-12	6.50E-271	5.90E-14	6.90E-16	3.40E-28	1.50	2.70E-02
N42-2	2.80E-01	9.80E-14	3.10E-13	3.20E-12	6.50E-271	5.90E-14	6.90E-16	3.40E-28	1.50	2.70E-02
KF147-2	1.40E-305	8.00E-21	3.70E-27	2.30E-20	1.10E-01	1.60E-01	6.70E-01	6.10E-20	0.28	3.40E-03
184-2	2.40E-305	7.90E-21	3.90E-27	2.10E-20	1.10E-01	1.60E-01	6.70E-01	6.10E-20	0.28	3.40E-03
G423	5.00E-306	1.70E-20	1.20E-26	1.90E-21	1.70E-01	3.10E-01	5.60E-01	1.60E-20	0.08	1.10E-03
M20-2	2.40E-306	1.70E-20	1.20E-26	1.90E-21	1.70E-01	3.10E-01	5.70E-01	1.60E-20	0.08	1.10E-03
KF67	2.40E-306	1.70E-20	1.20E-26	1.90E-21	1.70E-01	3.10E-01	5.70E-01	1.60E-20	0.08	1.10E-03
Li-1	1.10E-19	4.40E-43	3.40E-52	5.10E-289	5.70E-14	6.00E-08	6.80E-10	8.70E-30	0.88	5.50E-12
AI06	1.20E-19	4.80E-44	5.50E-53	7.90E-288	3.70E-14	4.90E-07	1.20E-09	5.00E-29	0.76	6.50E-12
M20-1	2.10E-18	7.10E-43	5.80E-53	1.00E-288	2.90E-13	5.30E-06	3.30E-08	7.20E-28	0.82	6.60E-12
JM1	2.60E-19	2.00E-42	1.90E-51	4.00E-289	6.00E-15	9.90E-09	3.00E-10	1.60E-30	0.80	1.70E-12
G50	5.70E-30	4.00E-82	7.10E-96	7.70E-54	6.90E-19	7.20E-15	3.80E-14	2.00E-27	1.00	1.50E-10
KF201	1.50E-16	1.00E-269	9.60E-113	1.90E-42	1.20E-12	4.10E-06	1.50E-10	5.30E-25	0.21	3.50E-09
IO-1	1.90E-18	4.90E-271	9.30E-114	3.20E-42	3.50E-13	2.80E-06	2.00E-11	2.70E-24	0.61	3.20E-08
CV56-1	2.10E-18	7.70E-272	9.60E-114	3.20E-42	9.10E-13	1.40E-05	4.30E-11	2.00E-24	0.69	1.80E-08
V4	2.10E-18	7.70E-272	9.60E-114	3.20E-42	9.10E-13	1.40E-05	4.30E-11	2.00E-24	0.69	1.80E-08
KF146-2	2.00E-16	1.80E-105	8.10E-140	1.50E-45	4.30E-16	1.60E-13	3.20E-18	4.30E-31	1.70	3.70E-04
N42-1	1.20E-18	4.60E-112	5.90E-182	7.40E-49	6.60E-16	6.40E-16	5.70E-16	2.00E-28	0.11	4.00E-05
CV56-2	2.50E-24	2.50E-113	7.70E-270	3.00E-51	8.30E-15	2.90E-14	5.80E-14	5.70E-29	0.00	N/A
A12	2.10E-23	2.30E-112	7.70E-270	1.80E-51	1.10E-14	5.40E-15	1.20E-14	2.10E-29	0.00	N/A

Figure SF2. phylogenetic tree and clade definition of the integrase function found in the *L. lactis* ICEs. The phylogenetic tree of the Int function multiple sequence alignment is displayed (left), combined with the E-values of each clade specific HMM profile on each entry (7-colour block in the middle), as well as the Pfam HMMs used to search for the lactococcal integrases that clearly classify all of them among the protein family PF00589 (three most right columns). Two integrase sequences appear to separate from the recognizable integrase clades that are represented by more than a single member. A clear distinction of the 7 recognizable clades can be discerned using the discriminatory HMMs, and the discrimination-boundary E-values. The two singleton integrases were not further analysed or discussed as a "clade" because their specific recognition would depend on the identification of more members of their clade. The colours used in this figure are the same as those used in Figure 5 of the main manuscript.

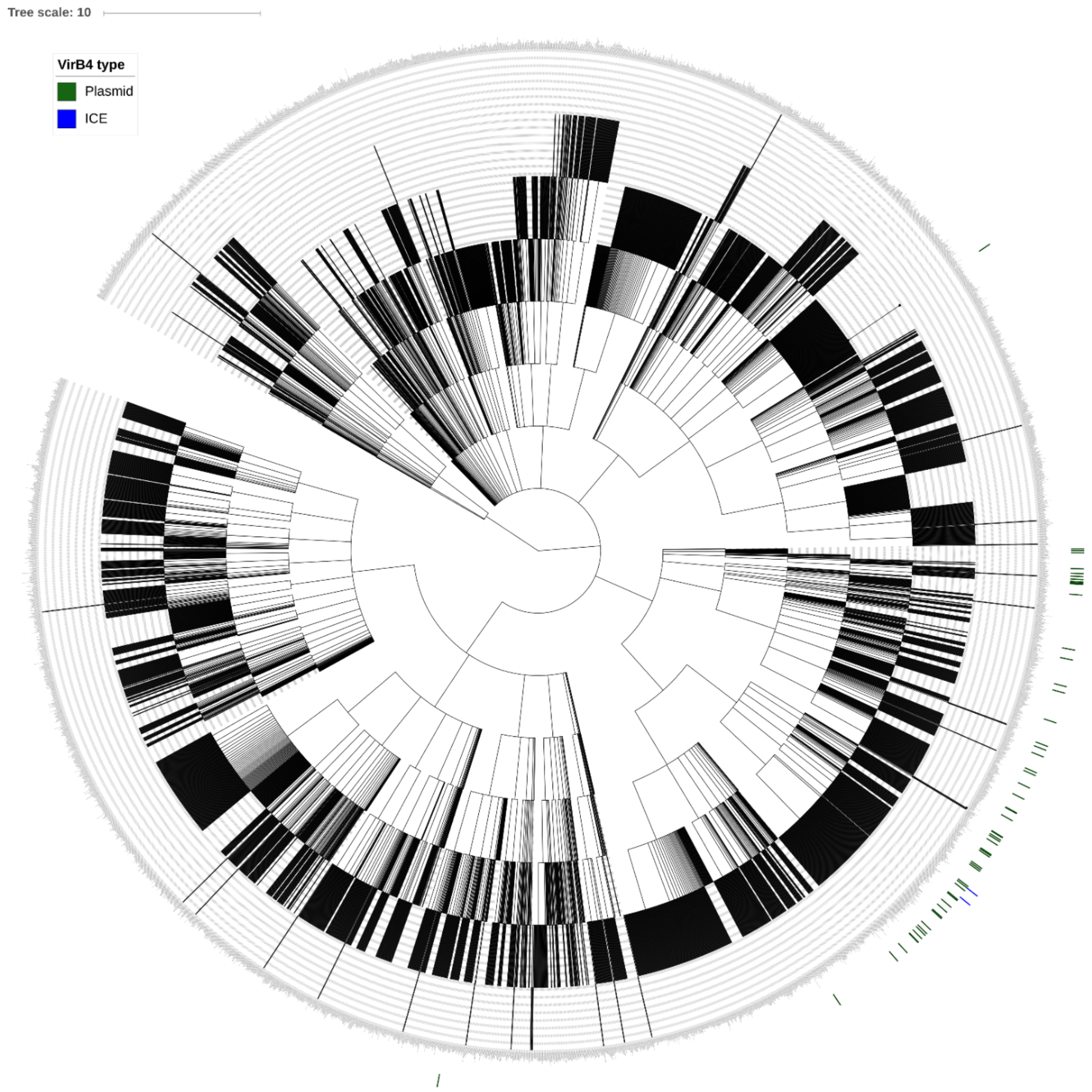

Supplemental figure SF3. Mapping of the detected virB4 proteins on the all the representative genomes. Two additional plasmid derived VirB4 hits outside of the Firmicutes can be seen in green.

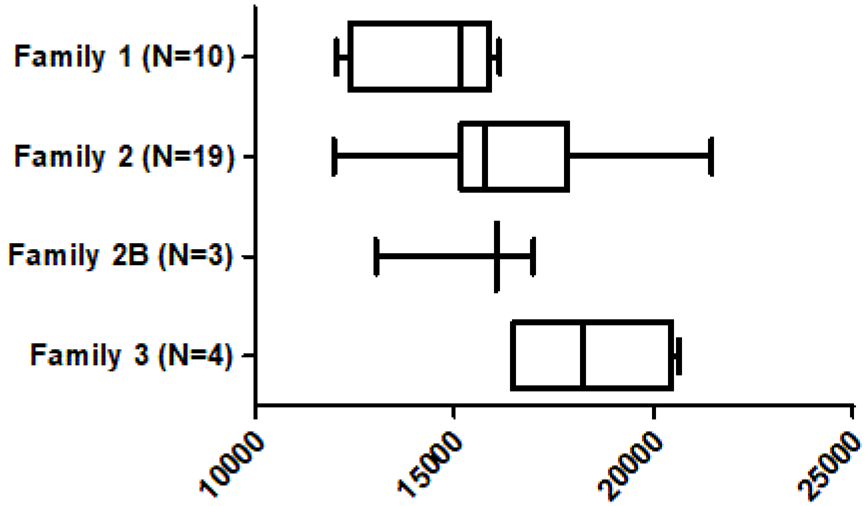

Supplemental figure SF4. Size distribution of the ICE core region, defined as integrase to coupling protein of each of the assigned ICE families. Family 2 shows the broadest size variation.
